# Instrument-Free and Visual Detection of *Salmonella* Based on Magnetic Nanoparticles and an Antibody Probe Immunosensor

**DOI:** 10.3390/ijms20184645

**Published:** 2019-09-19

**Authors:** Liding Zhang, Xuewei Du, Zhixin Chen, Congjie Chen, Nanxin Gong, Yihao Song, Yuzhu Song, Qinqin Han, Xueshan Xia, Haiming Luo, Jinyang Zhang

**Affiliations:** 1 Molecular Medicine Research Center of Yunnan Province, Faculty of Life Science and Technology, Kunming University of Science and Technology, 727 Jingming South Road, Kunming 650500, China; lidingzhang@aliyun.com (L.Z.); xueweidu@126.com (X.D.); arnoldchen1997@gmail.com (Z.C.); kmustccj@163.com (C.C.); gnxefforts@163.com (N.G.); ivan_syh@163.com (Y.S.); syzzam@126.com (Y.S.); qqhan10@kust.edu.cn (Q.H.); oliverxia2000@aliyun.com (X.X.); 2 Britton Chance Center for Biomedical Photonics, Wuhan National Laboratory for Optoelectronics-Huazhong University of Science and Technology, Wuhan 430074, Hubei, China; 3 MoE Key Laboratory for Biomedical Photonics, Collaborative Innovation Center for Biomedical Engineering, School of Engineering Sciences, Huazhong University of Science and Technology, Wuhan 430074, Hubei, China

**Keywords:** *Salmonella*, monoclonal antibody, magnetic nanoparticles, HRP-probe, immunoassay

## Abstract

*Salmonella*, a common foodborne pathogen, causes many cases of foodborne illness and poses a threat to public health worldwide. Immunological detection systems can be combined with nanoparticles to develop sensitive and portable detection technologies for timely screening of *Salmonella* infections. Here, we developed an antibody-probe-based immuno-N-hydroxysuccinimide (NHS) bead (AIB) system to detect *Salmonella.* After adding the antibody probe, *Salmonella* accumulated in the samples on the surfaces of the immuno-NHS beads (INBs), forming a sandwich structure (INB–*Salmonella*–probes). We demonstrated the utility of our AIB diagnostic system for detecting *Salmonella* in water, milk, and eggs, with a sensitivity of 9 CFU mL^−1^ in less than 50 min. The AIB diagnostic system exhibits highly specific detection and no cross-reaction with other similar microbial strains. With no specialized equipment or technical requirements, the AIB diagnostic method can be used for visual, rapid, and point-of-care detection of *Salmonella*.

## 1. Introduction

*Salmonella* is a common foodborne pathogen that infects humans and many other animals [1,2]. Cramps, diarrhea, vomiting, and fever are the most frequently reported symptoms of salmonellosis worldwide [3,4,5]. The elderly, the immunocompromised, and infants are the most commonly infected patients, experiencing significant morbidity and mortality [6,7]. It is estimated that approximately 94 million humans are infected with *Salmonella* globally each year, of which 80.3 million cases are foodborne [8,9,10]. Food is necessary for human survival, but it is often contaminated with *Salmonella*. The main carriers of *Salmonella* are poultry products, but other undercooked or raw meats, dairy products, and other industrialized foods are also easily contaminated with *Salmonella*, which can then infect humans [1]. Incidences of *Salmonella* infections have been reported in both developed and developing countries, and cases of *Salmonella* infections have increased in recent decades [11]. 

Due to the continuous persistence of pathogenic *Salmonella* infections, rapid point-of-care diagnostics are the first line of defense when an epidemic breaks out, and a rapid, sensitive, and point-of-care detection method is crucial for identifying and detecting *Salmonella*.

Recently, many methods have been reported for detecting *Salmonella*, including conventional culture methods and biochemical identification [12], polymerase chain reaction (PCR) [13], and loop-mediated isothermal amplification assay (LAMP) [14]. However, these methods either require a long pre-enrichment step or depend on specialized instruments and laboratory professionals [15]. Normal detection methods make it difficult to rapidly diagnose *Salmonella* without instruments. Thus, an instrument-free, rapid, and visual method remains to be developed. At present, many novelty biosensors are used for rapid and point-of-free detection of pathogenic microorganisms, including immunogold nanoparticles (IGNs) [16], lateral-flow strip immunoassays (LFSA) [17,18,19], and immunocapture loop-mediated isothermal amplification assay (IC-LAMP) [20,21]. However, both IGNs and LFSA are susceptible to either high pH or low pH and high salt ions, and have been associated with false positive results. Besides, the lack of ability to enrich the target substances from the environment means these methods lack high sensitivity. For some special samples, such as sputum, blood, and feces, some pretreatments are essential before detection. For IC-LAMP, the extraction of plasmids or genomes and portable high heat equipment are still needed before detection. More importantly, the aerosol produced by IC-LAMP leads to false positives.

On the contrary, immunomagnetic nanoparticles (IMNs) are new biological immunosensors that combine the magnetic beads with a special antibody. Compared with other detection methods based on nucleic acid or other sensors, the IMNs can realize the enrichment of bacteria without any special equipment or experimental skill. With the characteristics of rapid and high specificity of enrichment of the target substance isolated from the environment, it has been widely used for detection of viruses, bacteria, and toxins. Moreover, this technology, which does not require a long pre-enrichment step or extraction of genomes, is a time-saving and point-of-care method. 

However, there is no research reporting the detection of *Salmonella* based on immuno-N-hydroxysuccinimide (NHS) beads (INBs) and horseradish peroxidase (HRP) mAb probes at present. In this study, we firstly developed a novel immunosensor named the antibody-probe-based immuno-N-hydroxysuccinimide (NHS) beads (AIB) system to detect *Salmonella*. In this assay, we screened a pair of monoclonal antibodies (mAb) against *Salmonella*. First, mAb 2F1 was coated on the surface of 25-μm NHS-modified magnetic beads (NHS beads) to generate the INBs to capture *Salmonella*. The HRP mAb probes were generated using mAb 1B12 coupled with HRP. This system enabled the use of only a portable magnetic frame and 3, 3′, 5, 5′-tetramethylbenzidine (TMB) buffer to detect *Salmonella*, without requiring special equipment or skills. The advantages of the AIB system are the integration of both the specificity of antibodies and of INBs efficiently in pathogen cell enrichment, and it being more convenient, rapid, highly specific, and sensitive than other detection methods. Compared with carboxylic modified magnetic beads, the NHS beads used here were more efficiently coupled to antibodies without EDC or glutaraldehyde for activation. All of the process were performed within 1 h, saving about 17 h.

## 2. Results

### 2.1. Antibody-Probe-Based Immuno-N-Hydroxysuccinimide (NHS) bead (AIB) System Design

**Figure 1:**
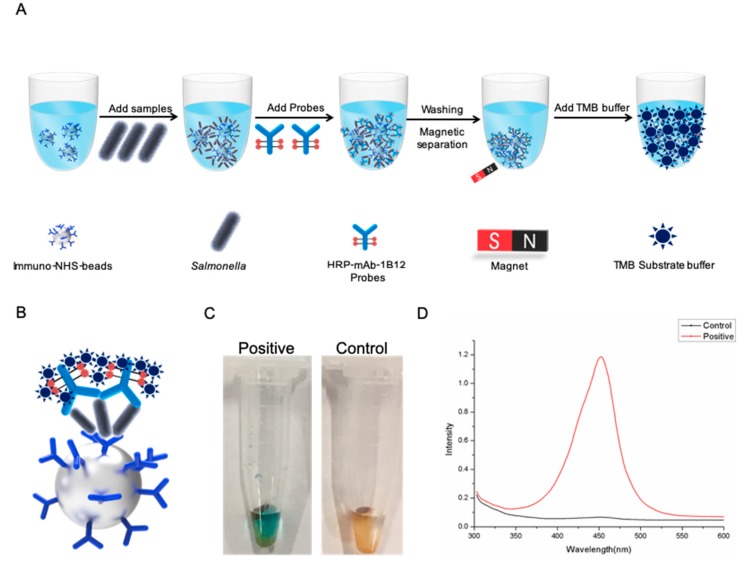
Schematic illustration of the AIB system for *Salmonella* detection. (**A**) *Salmonella* was captured by immuno-NHS beads (INBs), then labeled with specific HRP mAb 1B12 probes. The probe complexes changed color after addition of the TMB substrate solution. (**B**) Sandwich structure formed in the AIB system. (**C**) Images of the positive and control reactions in the AIB system. (**D**) Optical density of the positive and control reactions were recorded in the AIB system.

In this work, we developed an AIB system to rapidly and visually detect *Salmonella* using INBs and HRP mAb probes (Figure 1A). In the AIB system, mAb 2F1, which binds specifically with *Salmonella*, is coated on the surface of 25 μm NHS beads to form the INBs. When *Salmonella* appeared in the reaction, the INBs would capture it. Next, HRP mAb probes are added to the reaction, forming a sandwich structure (Figure 1B). Then, magnetic separation and washing are performed to remove the unbound probes. In the presence of TMB buffer, a positive reaction will be blue (Figure 1C), and the optical density (Figure 1D) will increase significantly.

### 2.2. Generation of Specific mAbs Against Salmonella

**Figure 2:**
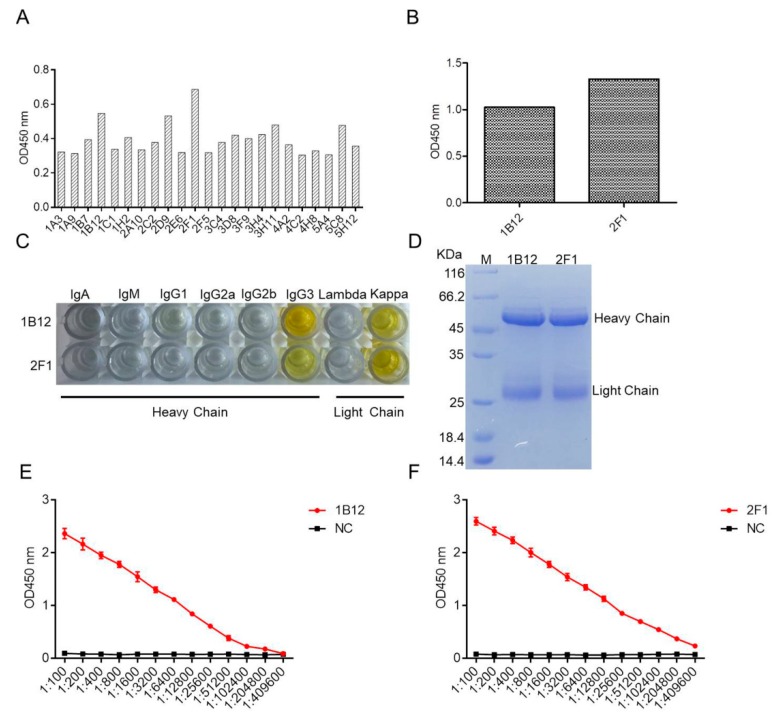
Generation and characterization of the mAbs against *Salmonella*. (**A**) OD values for each well after cell fusion were determined by ELISA. (**B**) OD values of 1B12 and 2F1 after three subcloning cycles were determined by ELISA. (**C**) Immunoglobulin isotypes of mAb 1B12 and 2F1. (**D**) Purified mAbs 1B12 and 2F1 were confirmed by SDS-PAGE. Titers of mAb 1B12 (**E**) and 2F1 (**F**).

Two stable positive hybridomas were screened through three subcloning cycles from twenty-three originally positive wells, designated as 2F1 and 1B12 (Figure 2A). Reactivity of the two mAbs was determined via enzyme-linked immunosorbant assay (ELISA). The results showed that both mAbs reacted with *Salmonella* (Figure 2B). The immunoglobulin isotypes of 2F1 and 1B12 were determined using a mouse monoclonal antibody isotyping kit. Figure 2C shows 2F1 and 1B12 isotyped as IgG3, and the light chains of the two mAbs belong to the kappa chain. The two mAbs were used to produce ascites. The ascites was purified using protein A-sepharose and tested via sodium dodecyl sulfate-polyacrylamide gel electrophoresis (SDS-PAGE) (Figure 2D). The titers of mAbs 2F1 and 1B12 were evaluated via ELISA, and the titers of both mAbs reached 1:204800 (Figure 2E,F). The K_D_ values of mAb 2F1 and 1B12 were measured as described in our previous published study [22], and were calculated as K_D_ = 3.677 ± 0.33 nM for mAb 2F1 and K_D_ = 1.126 ± 0.15 nM for mAb 1B12. 

### 2.3. Synthesis of the HRP mAb Probes

**Figure 3:**
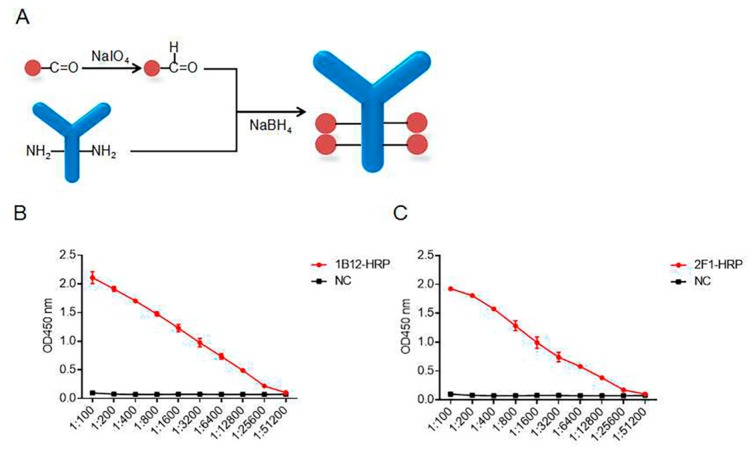
The mAb probe preparation and characterization. (**A**) Protocol for the mAb probe preparation. Titers of the HRP mAb 1B12 (**B**) and 2F1 (**C**) probes.

The purified mAbs 2F1 and 1B12 were dialyzed in phosphate-buffered saline (PBS) to remove the Tris-HCl and glycine. The purified mAbs and HRP were then coupled by an aldehyde–amino bridge under NaIO_4_ and NaBH_4_ (Figure 3A). The ratio of conjugation was calculated as 1 mg HRP/2.5 mg antibody. After conjugation, we acquired about 25 mg mAb probe containing 10 mg HRP. Next, the activities and titers of the two HRP mAb probes were determined by ELISA (Figure 3B,C). 

### 2.4. Characterization of the Paired Antibodies

**Figure 4:**
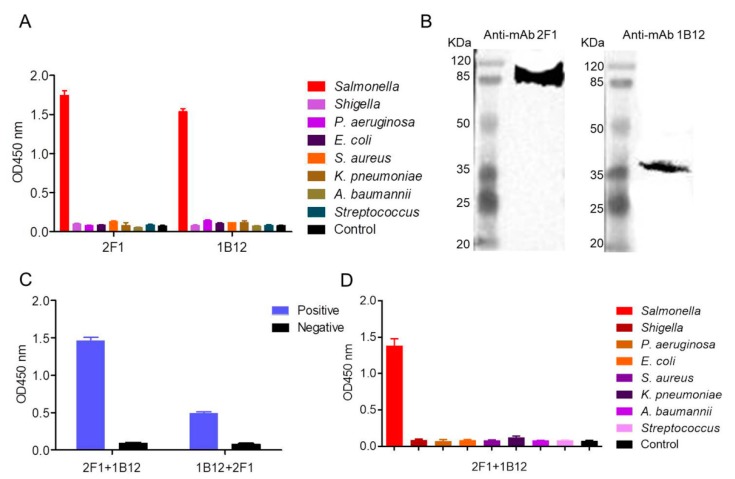
Establishment of the double sandwich ELISA (DAS-ELISA). (**A**) Specificity of mAbs 1B12 and 2F1 were determined by ELISA. (**B**) Western blot assay of the target proteins recognized by mAb 1B12 and 2F1. (**C**) Combinations tested with the DAS-ELISA. (**D**) Specificity of the 2F1+1B12 group based on the DAS-ELISA.

The reactivity and specificity of mAb 1B12 and 2F1 were evaluated via ELISA and Western blot. Figure 4A shows that both mAb 1B12 and 2F1 specifically recognized *Salmonella* and did not cross-react with similar microbial strains. The Western blot results showed that both mAbs recognized the different proteins on the surface of *Salmonella* (Figure 4B). The optimal mAbs used to establish the AIB system were screened based on double sandwich ELISA (DAS-ELISA). HRP-labeled mAbs (1B12 and 2F1) and unlabeled mAbs (1B12 and 2F1) were constructed in each group for the DAS-ELISA, which showed that the group composed of mAb 2F1 and 1B12 was more effective than the other groups (Figure 4C). In addition, we evaluated the specificity of the mAb 2F1 and 1B12 combination. Figure 4D shows that the group of composed of 1B12 and 2F1 displayed high specificity and did not recognize the control strains (*E. coli, S. aureus, K. pneumoniae, Shigella, A. baumannii, P. aeruginosa,* and *Streptococcus*). Figure 4C,D show that the mAb 2F1 and 1B12 group was the optimal combination for detection of *Salmonella*.

### 2.5. INB Preparation and Characterization

**Figure 5:**
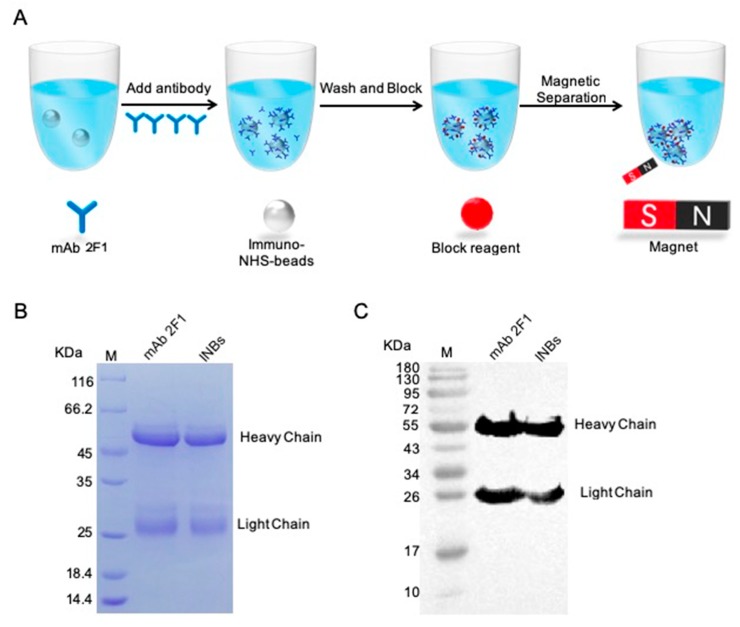
INB preparation and characterization. (**A**) Schematic of the INB preparation. (**B**) SDS-PAGE analysis of the INBs. (**C**) Western blot analysis of the INBs using goat anti-mouse IgG (H+L) HRP.

The INBs were generated by conjugating NHS modified magnetic beads (NHS beads) with mAb 2F1 via covalent coupling (Figure 5A). The prepared INBs were evaluated via SDS-PAGE and Western blot. The results demonstrated that mAb 2F1 had conjugated on the surface of the NHS beads (Figure 5B,C).

### 2.6. AIB System Optimization

**Figure 6:**
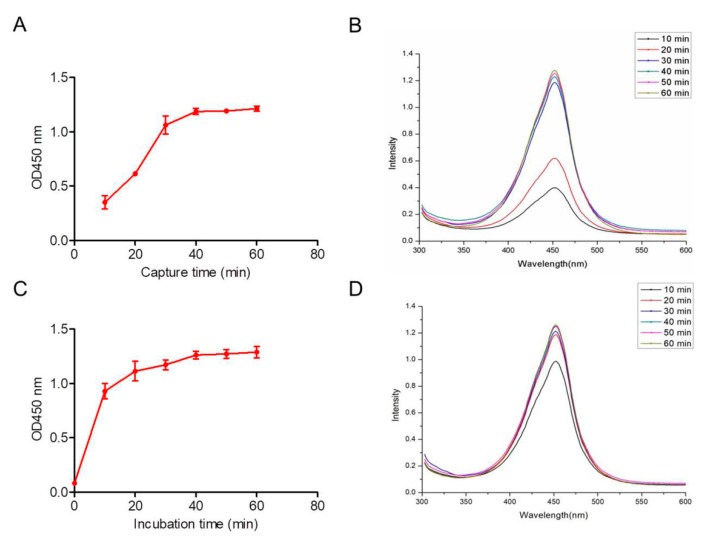
AIB system optimization. OD value (**A**) and optical density (**B**) of the time periods in which the INBs captured *Salmonella*. OD value (**C**) and optical density (**D**) of the time periods in which the mAb 1B12 probes formed the sandwich structure.

In the AIB system, we optimized each preparation step to achieve the best detection effect. Using gradient dilutions and plate counts, the average binding efficiency of the INBs was 90%. The optimum INB capture period was confirmed using various incubation times from 10 to 60 min. Figure 6A,B show that the INBs completely captured the *Salmonella* within 30 min. No significant differences were observed as the incubation time increased. The optimal incubation period, during which the mAb 1B12 probes formed sandwich products, was evaluated over 10 to 60 min (Figure 6C). The optical density from the formed sandwich products was determined via microplate reader. The sandwich products quickly formed within 20 min, demonstrating the high affinity between the mAb 1B12 probes and *Salmonella* (Figure 6D).

### 2.7. Assessment of Salmonella Detection Using the AIB System

**Figure 7:**
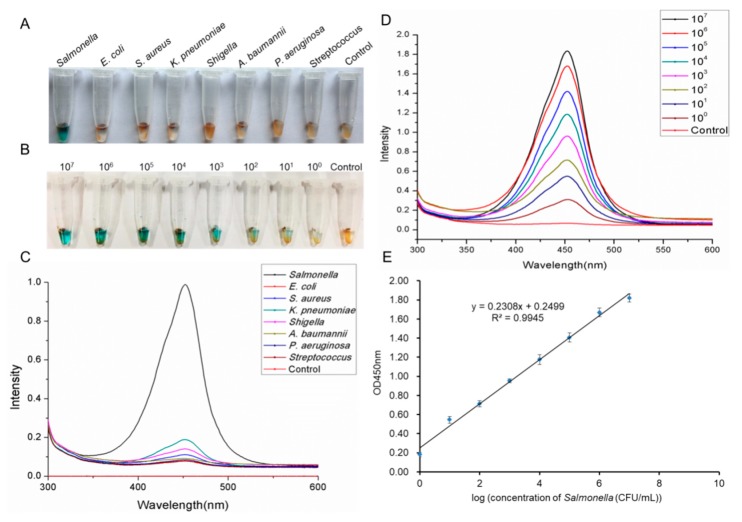
Evaluation of the AIB system. Specificity assay of the AIB system for *Salmonella* detection: (**A**) images and (**C**) optical density of *Salmonella* and non-target strains in the AIB system. Sensitivity assay of the AIB system for *Salmonella* detection: (**B**) change in color and (**D**) optical density due to different concentrations of *Salmonella* (from 10^7^ CFU mL^−1^ to 10^0^ CFU mL^−1^). (**E**) Plotted linear curve of the AIB system with *Salmonella* ranging from 10^7^ to 10^0^ CFU mL^−1^.

We initially tested the AIB system specificity using seven similar microbial strains and found that the AIB system was highly specific for detecting *Salmonella*. The blue color (Figure 7A) and strong optical density (Figure 7C) were observed only in the presence of *Salmonella*. The controls showed no significant changes. The AIB system sensitivity was tested with different *Salmonella* concentrations ranging from 9 × 10^7^ to 9 × 10^0^ colony-forming units (CFU) mL^−1^. Figure 7B,D show the blue color and optical density at different *Salmonella* concentrations, as recorded by the AIB system. We then set up a plotted linear curve using the different *Salmonella* concentrations. Figure 7E shows a good linear relationship (R^2^ = 0.9945).

### 2.8. Salmonella Detection by the AIB System in Artificially Contaminated Samples 

**Figure 8:**
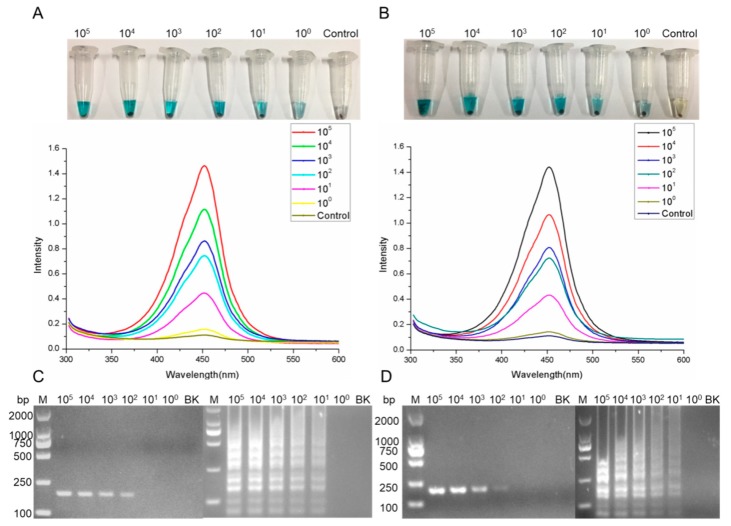
*Salmonella* detection in milk and egg samples using the AIB system. *Salmonella* detection in milk and egg samples with *Salmonella* concentrations from 10^5^ to 10^0^ CFU mL^−1^. The blue color and images of the optical density recorded in the milk (**A**) and egg (**B**) samples. The PCR and LAMP assays applied for the detection of *Salmonella* in milk samples (**C**) and egg samples (**D**). Abbreviations: BK = blank control.

To evaluate the AIB system performance, we used milk and egg samples contaminated with different concentrations of *Salmonella* ranging from 10^5^ to 10^0^ CFU mL^−1^. Figure 8A,B show the changes in blue color and strong optical density in the milk and egg samples contaminated with different *Salmonella* concentrations. These results confirmed that the new AIB system can rapidly and accurately detect *Salmonella*, even in complex samples such as milk and eggs. Besides, the prepared milk and egg samples were used for the extraction of the genome and then applied for PCR and LAMP assays using the reported primers [23]. Figure 8C,D show that the sensitivity values of PCR and LAMP were 10^2^ CFU and 10^1^ CFU, respectively, in both milk and egg samples.

## 3. Discussion

*Salmonella* is an important human pathogen worldwide, infecting humans and various other animals. Improper cooking and processing of animal-derived foods (e.g., raw milk, meat, and eggs) are the main mechanisms by which *Salmonella* infects humans [24]. Salmonellosis, one of the most important zoonoses, which mainly causes severe foodborne gastroenteritis and bacterial diarrhea, is a huge public health problem [25]. Foods such as meat, milk, and eggs are essential for human survival but are often contaminated with *Salmonella* [24]. Studies have reported that cases of *Salmonella* poisoning have occurred in powdered infant formula [26], raw milk [27], eggs [28], and meat [13,29]; thus, the food industry must prioritize developing innovative methods for detecting *Salmonella*.

Conventional culture-based methods are considered the gold standard for detecting *Salmonella* in various samples. However, these are labor-intensive and time-consuming, usually taking 2 to 3 days [30]. Thus, these methods are unsuitable for rapid detection. Recently, rapid detection methods have been developed to detect *Salmonella* that target the nucleic acid, including PCR [13], real-time PCR [31], immunocaptured-PCR (IC-PCR) [32], and LAMP [8,33]. The disadvantages of PCR and IC-PCR detection methods are that they require instruments, professional personnel, bacterial enrichment, and genome or plasmid extraction [15,34]. LAMP is a novel amplification approach developed by Notomi et al. [35], which is rapid and highly specific, and has been applied for various pathogens, including parasites [36,37], fungi [38], bacteria [20], and viruses [39,40]. The biggest disadvantage of LAMP is that it produces aerosol during detection, leading to many false positives. 

Conversely, immunodiagnostic approaches are more rapid, sensitive, and stable than nucleic acid assays, especially for samples such as milk [41], whole blood [42], and saliva [43]. Many novel immunosensors have been developed, which combine immunology with magnetic nanoparticles [22], platinum nanoparticles [44], or Pt nanomotors [45]. Instrument-free and mobile diagnostic technologies could transform the current foodborne pathogen detection systems, particularly in resource-limited settings.

In this study, we developed an instrument-free, sensitive immunosensor to effectively, rapidly, and sensitively detect *Salmonella* based on a pair of mAbs recognizing different antigenic determinants on the surface of *Salmonella* (Figure 4B) and sensitive probes (Figure 3). The AIB system reported in this manuscript was developed using a mAb pair, NHS beads, and HRP. In this system, mAb 1B12 was conjugated with HRP, forming a sandwich structure (Figure 3). The mAb 2F1 was coated on the surface of the NHS beads used to enrich *Salmonella* (Figure 5). We demonstrated the feasibility and practicability of the AIB system for detecting *Salmonella* using INBs and HRP probes. The sensitivity of the AIB system was 9 CFU mL^−1^ and higher than that of PCR (10^5^ CFU) [13], real-time PCR coupled with immunomagnetic separation or centrifugation (2 × 10^4^ CFU) [42], LAMP (1.3 to 28 CFU) [43], conventional culture-based methods, and antibody or aptamer-based assay (10^1^ to 10^3^ CFU) (Table 1). However, the nucleic-acid based detection methods still take several hours to enrich the *Salmonella*, followed by extraction of the plasmid or genome using a commercial kit [13,31,46]. Besides, special equipment, including the thermal cycling instrument, an electrophoresis apparatus, and a gel imaging system, are essential for PCR [13] and real-time PCR [31,32] methods. This special equipment is expensive and requires professional technical assistance, which largely limits their applications, especially for areas with poor resources. LAMP is a simple detection method that does not require special equipment (unlike PCR or real-time PCR), but it cannot enrich the target substances from the samples, and a commercial kit is also needed to extract the plasmid or genome [37,38]. Moreover, for some special samples, such as sputum, blood, and feces, it is very difficult to accurately and quickly enrich the target substances, and much time and materials are needed during the process [47]. Additionally, the false positive results caused by LAMP can be deadly. Therefore, the above drawbacks make LAMP inappropriate for fast, sensitive on-site detection. For antibody or aptamer-based ELISA assay, the sensitivity of the related ELISA assay ranged from 10^1^ to 10^3^ CFU for artificially samples, but the process takes several hours. In addition, similar INB methods, ELISA assay lacks the ability to rapidly enrich targets from the environment, especially for special samples such as milk, whole blood, and saliva, meaning antibody or aptamer-based ELISA assays are insufficiently sensitive, which largely limits their applications. Besides, professional experimental skills are essential, as well as a microplate reader, which is expensive and not available anywhere, particularly in resource-poor areas.

Compared with culture-based techniques and biochemical assays, PCR, real-time PCR, and normal LAMP, and antibody or aptamer-based ELISA assay, the AIB assay only needs a portable magnetic frame and TMB buffer. It does not require a long pre-enrichment step followed by genome or plasmid extraction, any special equipment, or professional skills. The AIB system developed here has two advantages over normal detection methods. The first one is that the surfaces of the NHS beads are coated with abundant mAb 2F1 that specifically recognize *Salmonella* (Figure 4A), which means the prepared INBs can efficiently and accurately enrich the *Salmonella* from different biological samples within 30 min, as shown in Figure 6A,B. Compared with the carboxylic modified magnetic beads used in our previous study and in other research articles [22], the NHS beads are coupled with antibodies with high efficiency and short timeframes, without needing EDC or glutaraldehyde for activation. The other advantage is that the mAb probe, having high affinity, can rapidly form a sandwich structure within 20 min, as shown in Figure 6C,D. Additionally, the HRP coupled with mAb 1B12 is efficiently catalyzed by TMB buffer, showing a strong blue color (Figure 1C). The optical density (Figure 1D) changed within 10 min. Accordingly, the INBs showing rapid and efficient enrichment together with the sensitive mAb probe allow the AIB system to rapidly and sensitively detect *Salmonella* within 50 min. Figure 7 shows that the AIB system has good sensitivity and specificity based on the INBs and HRP mAb probes. We evaluated the practical application of the AIB system using artificially contaminated samples, including water (Figure 7B,D), milk (Figure 8A), and eggs (Figure 8B). The results indicated that the AIB system enabled accurate and rapid screening of *Salmonella* and could potentially be used in the food industry and hospitals. The prepared samples were also identified by PCR and LAMP to evaluate the accuracy of the AIB assay. In Figure 8C,D, we can clearly see that the minimum detection limits for PCR and LAMP were 9 × 10^2^ CFU and 9 × 10^1^ CFU, respectively, in both milk and eggs samples. The sensitivity of the PCR and LAMP methods was lower than the AIB assay for the same samples. Having high sensitivity and specificity, the AIB system can rapidly and accurately detect *Salmonella* without requiring special equipment or professional skills, making the AIB system more applicable in various environments. 

## 4. Materials and Methods 

### 4.1. Materials 

Table 2 shows the eight bacterial strains used in this study. The SP2/0 cells were stored in our lab. Freund’s incomplete adjuvant (FIA), Freund’s complete adjuvant (FCA), hypoxanthine–aminopterin–thymidine (HAT), hypoxanthine–thymidine (HT), and PEG-2000 were purchased from Sigma (St. Louis, MO, USA). The fetal bovine serum (FBS) and Roswell Park Memorial Institute (RPMI) 1640 medium were purchased from Gibco (Grand Island, NY, USA). NHS beads and the MAg25K/NHS kit were purchased from Enriching Biotechnology (Suzhou, China). Protein A-sepharose was purchased from GE Healthcare (Chicago, IL, USA). The mouse monoclonal antibody isotyping kit was purchased from Southern Biotech (Birmingham, AL, USA). Horseradish peroxidase (HRP) and goat anti-mouse IgG (H+L) HRP were purchased from GenScript (Nanjing, China).

### 4.2. Production of mAbs Against Salmonella

The antigens were prepared using 10^8^ CFU of *Salmonella* dissolved in PBS buffer and inactivated for 30 min at 80 °C [20]. The prepared antigens were used to immunize BALB/c mice, with 10^7^ CFU being mixed with FIA for the first immunization and 10^7^ CFU mixed with FCA for the second and third immunizations. Three days after the boosted immunization, spleen cells were collected and fused with SP2/0 cells via PEG at 37 °C [79]. The fused cells were maintained in HAT medium for 7 days. One week later, the fused cells were cultured in HT medium until the first round of screening. The positive hybridomas in each plate were screened by ELISA.

### 4.3. Purification of Ascites

After three subcloning cycles, we successfully obtained two positive hybridoma cell lines that stably secreted antibodies against *Salmonella*. Two hybridomas were constructed to prepare the ascites, which were purified using protein A-sepharose. Briefly, all ascites were filtered using a 0.2-micron filter, and the pH value of the ascites was adjusted to 8.0 using 1.0 M Tris-Cl (pH 8.0). Next, the prepared ascites were incubated with protein A-sepharose for 30 min at 25 °C. After incubation, the ascites were collected, and the column was washed with 100 mM Tris-Cl (pH 8.0) and 10 mM Tris-Cl (pH 8.0), respectively. Lastly, the column was washed with 50 mM glycine (pH 3.0), and the purified immunoglobulins from each antibody were determined via bicinchoninic acid assay and SDS-PAGE.

### 4.4. ELISA and Western Blot Assay

For the ELISA, each well was coated with 10^6^ CFU of *Salmonella* and incubated for 2 h at 37 °C. All wells were then washed with PBS-T and blocked with 5% BSA. Next, mAb 1B12 and 2F1 (1:1000) were added to each well and incubated for 1 h at 37 °C. All wells were washed with PBS-T, then the goat anti-mouse IgG (H+L) HRP (1:5000) was added and incubated for 1 h at 37 °C. One hour later, the supernatant was removed and washed with PBS-T. Lastly, soluble TMB substrate solution (TIANGEN, Beijing, China) was added to detect the immunoreaction. 

For the Western blot assay, 10^6^ CFU of *Salmonella* was transferred to a nitrocellulose (NC) membrane, which was blocked with 5% BSA for 1 h at 37 °C. Next, mAb 1B12 and 2F1 were added (1:1000) and incubated for 2 h at 37 °C. Two hours later, the antibodies were collected and the NC membranes were washed with PBS-T. Goat anti-mouse IgG (H+ L) HRP (1:5000) was then added and incubated for 1 h at 37 °C. Lastly, the NC membranes were washed, and the Western blot kit (BioBest, Anhui, China) was used to detect the immunoreaction. 

### 4.5. HRP mAb Probe Preparation and Characterization

The HRP mAb probes were synthesized using mAbs coupled with HRP. All probes were prepared as described in our previous study [22]. Briefly, 10 mg of HRP was dissolved in 1 mL of 0.1 M NaHCO_3_ and then oxidized for 2 h with 1 mL of 10 mM NaIO_4_. Then, the HRP liquid was mixed with 1.5 mL of 0.1 M Na_2_CO_3_, 25 mg of 1B12, 2F1, and reacted in a 5 mL tube containing 0.6 g of Sephadex G25 for 3 h at 25 °C. Three hours later, the liquid was collected and terminated with 0.225 mL of 0.132 M NaBH_4_ for 0.5 h, followed by 0.675 mL of 0.132 M NaBH_4_ for 1 h at 25 °C. The process was performed in the dark. Lastly, the prepared HRP mAb probes were stored in PBS buffer at 4 °C. The activities and titers of the HRP mAb probes were determined using ELISA. Briefly, two HRP mAb probes at different dilutions (from 1:100 to 1:51200) were added to each well, which had been coated with *Salmonella* and blocked with BSA, and incubated for 1 h at 37 °C. Next, the supernatant was removed from each well and washed with PBS-T. The immune responses were then detected using soluble TMB substrate solution.

### 4.6. INB Preparation and Characterization

The INBs were prepared using NHS beads conjugated with mAb 2F1 using a MAg25K/NHS kit. First, the NHS beads were mixed evenly, and 1 mL of NHS bead solution (10%, v/v) was added to a 2 mL tube. The supernatant was removed by magnetic separation, and the NHS beads were washed twice with 2 mL of absolute ethanol. Second, 1.2 mg of mAb 2F1 dissolved in a coupling buffer was added and mixed with NHS beads for 2 h at room temperature. Next, 1 mL of blocking buffer was added to block the beads for 2 h, while being rotated and mixed at room temperature. After blocking, the supernatant was removed, and 1 mL of wash buffer was added to wash the beads five times. Lastly, the supernatant was removed, and the INBs were dissolved in PBS buffer (pH 7.4) for further use. The prepared INBs were evaluated via SDS-PAGE, Western blot, and the AIB system.

### 4.7. Performance of the AIB System 

The sensitivity of the AIB system was evaluated using serially diluted *Salmonella*, with concentrations ranging from 10^7^ to 10^0^ CFU mL^−1^. One microliter of each *Salmonella* concentration was tested using the AIB system following the testing protocol. The specificity of the developed AIB system was confirmed using *Salmonella* and similar microbial strains (*E. coli*, *S. aureus*, *K. pneumoniae*, *Shigella*, *A. baumannii*, *P. aeruginosa*, and *Streptococcus*).

### 4.8. System Evaluation using Contaminated Samples

To evaluate the AIB system, we used artificial milk and egg samples containing *Salmonella* concentrations ranging from 10^5^ to 10^0^ CFU mL^−1^. Each sample was tested using the AIB system according to the testing protocol.

## 5. Conclusions

In this study, we developed a novel immunosensor based on magnetic NHS beads and antibody probes. The novel AIB system enabled visual, rapid, and sensitive *Salmonella* detection, without requiring specialized equipment or skills. This system can potentially be widely used to diagnose infectious diseases caused by *Salmonella* spp.

## Figures and Tables

**Table 1:** Comparison between AIB, PCR, LAMP, real-time PCR, IC-PCR, ELISA, PCR, or LAMP-ELISA, and DNA aptamer assay.

Results	Methods							
	AIB	PCR [13,15,47,48,49]	LAMP [9,14,50,51,52]	Real-Time PCR [31,32,46,53,54,55,56]	IC-PCR [46,57,58,59]	ELISA [60,61,62,63,64,65]	PCR [66,67,68,69], LAMP-ELISA [70]	DNA Aptamer Assay [71,72,73,74,75,76,77,78]
Sensitivity	9 CFU for artificial sample	10^2^ to10^5^ CFU for artificial sample	1.3 to 28 CFU for artificial sample	10^2^ to 10^4^ CFU for artificial sample	10^2^ to 10^3^ CFU for artificial sample	10^2^ to 10^3^ CFU for artificial sample	10^1^ to 10^3^ CFU for artificial sample	10^1^ to 10^3^ CFU for artificial sample
Need times	50 min	14 h	3 h	1 h to 8 h	1.5 h	8 h to 23 h	8 h to 23 h	3 h to 23 h
Equipment	Magnet, TMB buffer	Bacterial enrichment, genomic extraction kit, PCR equipment and related reagents, DNeasy	Bacterial enrichment, genomic extraction kit, LAMP equipment and related reagents	Bacterial enrichment, genomic extraction kit, real-time PCR equipment and related reagents	Magnet, genomic extraction kit, real-time PCR equipment and related reagents	96-well plates, antibodies, PBS-T, TMB buffer	PCR or LAMP equipment and related reagents, 96-well plates, antibodies, PBS-T, TMB buffer	Aptamers, PBS-T, PBS, centrifuge, TMB buffer

**Table 2:** Bacterial strains used in this study.

Bacterial	Bacterial Strains Source
*Salmonella*	Isolate from monkey
*Salmonella*	ATCC13076
*Shigella*	Isolate from monkey
*A. baumannii*	Isolate from clinical samples
*P. aeruginosa*	Isolate from secretion substance
*E. coli*	ATCC25922
*K. pneumoniae*	Isolate from clinical samples
*S. aureus*	ATCC29213
*Streptococcus*	Isolate from clinical samples

## Abbreviations

AIB	antibody-probe-based immuno-N-hydroxysuccinimide beads
DAS-ELISA	double sandwich ELISA
ELISA	enzyme-linked immunosorbant assay
FBS	fatal bovine serum
FCA	Freund’s complete adjuvant
FIA	Freund’s incomplete adjuvant
IC-PCR	immunocaptured-PCR
INBs	immuno-NHS beads
LAMP	loop-mediated isothermal amplification assay
mAb	monoclonal antibody
NHS beads	N-hydroxysuccinimide (NHS) beads
PCR	polymerase chain reaction
HAT	hypoxanthine–aminopterin–thymidine
HRP	horse radish peroxidase
HT	hypoxanthine–thymidine
SDS-PAGE	sodium dodecyl sulfate-polyacrylamide gel electrophoresis
TMB	3, 3′, 5, 5′-tetramethylbenzidine
